# Molecular Basis for Involvement of CYP1B1 in MYOC Upregulation and Its Potential Implication in Glaucoma Pathogenesis

**DOI:** 10.1371/journal.pone.0045077

**Published:** 2012-09-21

**Authors:** Suddhasil Mookherjee, Moulinath Acharya, Deblina Banerjee, Ashima Bhattacharjee, Kunal Ray

**Affiliations:** Molecular & Human Genetics Division, CSIR-Indian Institute of Chemical Biology, Kolkata, India; Sudbury Regional Hospital, Canada

## Abstract

*CYP1B1* has been implicated in primary congenital glaucoma with autosomal recessive mode of inheritance. Mutations in *CYP1B1* have also been reported in primary open angle glaucoma (POAG) cases and suggested to act as a modifier of the disease along with *Myocilin* (*MYOC*). Earlier reports suggest that over-expression of myocilin leads to POAG pathogenesis. Taken together, we propose a functional interaction between CYP1B1 and myocilin where 17β estradiol acts as a mediator. Therefore, we hypothesize that 17β estradiol can induce *MYOC* expression through the putative estrogen responsive elements (EREs) located in its promoter and CYP1B1 could manipulate *MYOC* expression by metabolizing 17β estradiol to 4-hydroxy estradiol, thus preventing it from binding to *MYOC* promoter. Hence any mutation in *CYP1B1* that reduces its 17β estradiol metabolizing activity might lead to *MYOC* upregulation, which in turn might play a role in glaucoma pathogenesis. It was observed that 17β estradiol is present in Human Trabecular Meshwork cells (HTM) and Retinal Pigment Epithelial cells (RPE) by immunoflouresence and ELISA. Also, the expression of enzymes related to estrogen biosynthesis pathway was observed in both cell lines by RT-PCR. Subsequent evaluation of the EREs in the *MYOC* promoter by luciferase assay, with dose and time dependent treatment of 17β estradiol, showed that the EREs are indeed active. This observation was further validated by direct binding of estrogen receptors (ER) on EREs in *MYOC* promoter and subsequent upregulation in *MYOC* level in HTM cells on 17β estradiol treatment. Interestingly, *CYP1B1* mutants with less than 10% enzymatic activity were found to increase the level of endogenous myocilin in HTM cells. Thus the experimental observations are consistent with our proposed hypothesis that mutant CYP1B1, lacking the 17β estradiol metabolizing activity, can cause MYOC upregulation, which might have a potential implication in glaucoma pathogenesis.

## Introduction

Glaucoma is a multifactorial optic disc neuropathy in which there is characteristic acquired loss of retinal ganglion cells and atrophy of the optic nerve [1]. It is the second largest blinding disorder after cataract [2]. According to the latest estimates, worldwide 5.7 million people are visually impaired and about 3.1 million people are blind due to glaucoma [2].

Among the various glaucoma subtypes, primary open angle glaucoma (POAG) occurs most frequently. Transmission of the disease occurs mostly in monogenic form in juvenile onset POAG (JOAG) and complex form in adults. The complex nature of the POAG has been reviewed recently [3]. It has been reported that 72% of POAG cases have an inherited component [4]. Thirty three chromosomal loci have so far been implicated in POAG, of which four genes, *Myocilin* (*MYOC*) on *GLC1A* (1q32) [5], *Optineurin* (*OPTN*) [6] on *GLC1E* (10p25), *WDR36* on *GLC1G* (5q22.3) [7] and *NTF4* on *GLC1O* (19q13.3) [8], [9] have been characterized. In most cases, however, in spite of clear familial clustering, POAG does not follow a Mendelian pattern of inheritance. All studies carried out on the role of *MYOC* in POAG including the largest study done on 1703 patients [10] reported similar frequency (2–4%) of mutations in *MYOC*.

In 1997, *CYP1B1* was first identified as a causal gene for primary congenital glaucoma (PCG) [11]. Later, it was reported as a modifier locus for POAG that together with *MYOC* mutation expedite the disease progression from adult onset to a juvenile form in a digenic mode of inheritance [12]. Screening *CYP1B1* in 236 unrelated French Caucasian POAG patients unraveled mutations in 4.6% (n = 11) of the patients with no mutation in *MYOC* [13]. We observed that on rare occasion even *CYP1B1* alone could be responsible for JOAG [14].

CYP1B1 is a multifactorial enzyme involved in fatty acid, retinoic acid and 17β estradiol metabolism. Multiple studies have demonstrated that mutations in *CYP1B1* results in the loss of one or more of its enzymatic activity, stability and relative abundance [15]–[19] but no studies have been done yet to determine the mechanism operating in digenic scenarios in POAG cases involving both *CYP1B1* and *MYOC* mutations. We here propose a mechanism based on our experimental data that could potentially explain monogenic as well as digenic association of *CYP1B1* along with *MYOC* in POAG. We hypothesize that CYP1B1 can manipulate myocilin expression by metabolizing 17β estradiol to 4-hydroxy estradiol in Human Trabecular Meshwork (HTM) cells preventing it from binding the Estrogen Receptors (ER) present in cells and thus limiting the activation of the putative EREs present in the *MYOC* promoter region. Mutant CYP1B1, lacking 17β estradiol metabolizing activity, may lead to accumulation of higher level of 17β estradiol in cells, thus leading to prolonged activation of the EREs in the *MYOC* promoter region via ERs and upregulate myocilin expression by transcriptional activation. We attempted to prove this hypothesis by providing supporting evidence for the following biological events: (1) 17β estradiol is present in HTM cells and is synthesized in the trabecular meshwork itself; (2) the EREs in the *MYOC* promoter region are active; (3) 17β estradiol via EREs can cause transcriptional activation of *MYOC* by nuclear localization of ERα and direct binding of ERα-17β estradiol complex to the EREs; and (4) specific CYP1B1 mutants, lacking 17β estradiol metabolizing activity, can upregulate endogenous myocilin in HTM cells.

## Results

### 17β Estradiol is Present in HTM and RPE Cells

**Figure 1:**
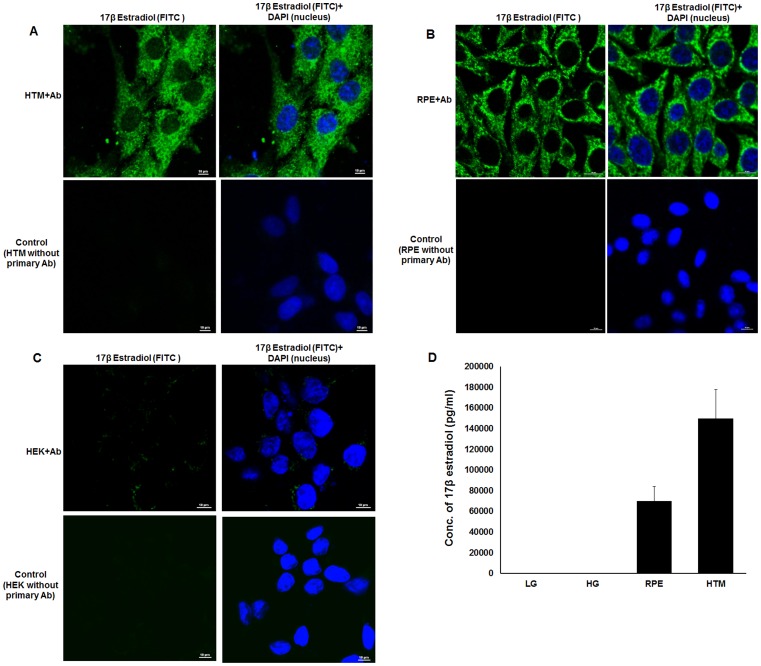
Presence of 17β estradiol in ocular cells. Confocal images are shown for HTM (*Panel A*), RPE (*Panel B*) and HEK 293 (*Panel C*) cells using anti-17β estradiol antibody and counterstained with FITC labeled secondary antibody. DAPI was used to stain the nucleus. In each panel control cells were treated only with FITC labeled secondary antibody, but not primary antibody, to assess the background noise. The scale of magnification is shown in each panel. The level of 17β estradiol in HTM and RPE cell lines were estimated by ELISA (*Panel D*). Similar estimation in low glucose (LG) and high glucose (HG) media containing 10% charcoal treated FBS did not show presence of 17β estradiol. The experiments were done in triplicate.

To probe the presence of 17β estradiol in ocular cells, HTM and RPE cells were grown approximately for 36 hours in culture media and treated with 17β estradiol antibody followed by FITC conjugated anti-mouse secondary antibody. Immunofluoresence assay showed scattered expression of 17β estradiol in the cytosol of HTM (Figure 1A) and RPE (Figure 1B) that was absent in the negative control (cells treated only with FITC conjugated anti-mouse secondary antibody). To eliminate the possibility of experimental artifact due to non-specific binding, same experiment was performed with HEK293 (Human Embryonic Kidney) cells that revealed the presence of 17β estradiol at a very low level in these cells (Figure 1C).

Under normal condition, these cells were grown in a media containing FBS. Thus these cells might have contaminating amount of the hormone carried by FBS used in cell culture. In order to eliminate such a possibility, HTM and RPE cells were grown for several passages in media supplemented with charcoal treated FBS to remove 17β-estradiol. Both cell types as well as the culture media were treated with di-ethyl ether to extract 17β-estradiol. The concentration of the extracted estradiol was determined from the standard curve and was found to be 70,000 pg/ml and 150,000 pg/ml in HTM and RPE cell lines respectively and the media was found to contain ≤200 pg/ml 17β estradiol (Figure 1D). These observations strongly suggested the presence of 17β estradiol in HTM and RPE cells.

### Presence of Estrogen Biosynthesis Pathway Enzymes in HTM and RPE Cells

**Figure 2:**
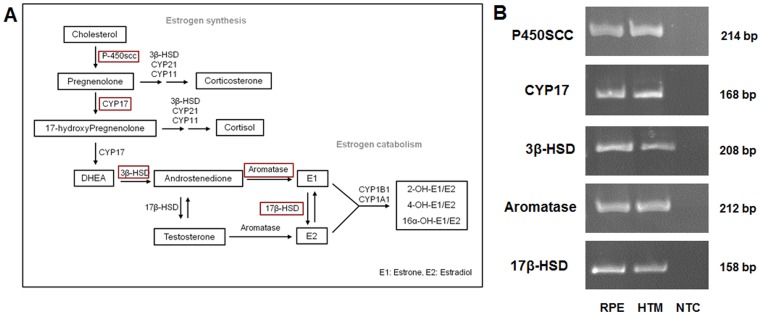
Expression of 17β estradiol synthesizing enzymes in HTM and RPE cells. *A*: 17β estradiol synthesis pathway. The key enzymes are highlighted by red squares. ***B*** **:** Semi-quantitative RT-PCR showing the presence of key 17β estradiol synthesizing enzymes in HTM and RPE cells. Three independent experiments were done for each enzyme in both cell lines. The identity of each product was confirmed by sequencing (data not shown). NTC: No cDNA template control.

To gather additional support in favor of intracellular synthesis of 17β estradiol in HTM and RPE, we examined for the presence of the enzymes involved in the biosynthetic pathway of estradiol from cholesterol in these cell lines. In both HTM and RPE cells, expression of *P450SCC*, *CYP17*, *3β-HSD*, *17β-HSD* and *Aromatase* (Figure 2A) were found by RT-PCR (Figure 2B) using gene specific primer pairs that encompass an intron to distinguish the amplified products from genomic sequences. The PCR products were further sequenced to confirm the identity of the target region (data not shown).

### EREs in the *MYOC* Promoter are Functionally Active

**Figure 3:**
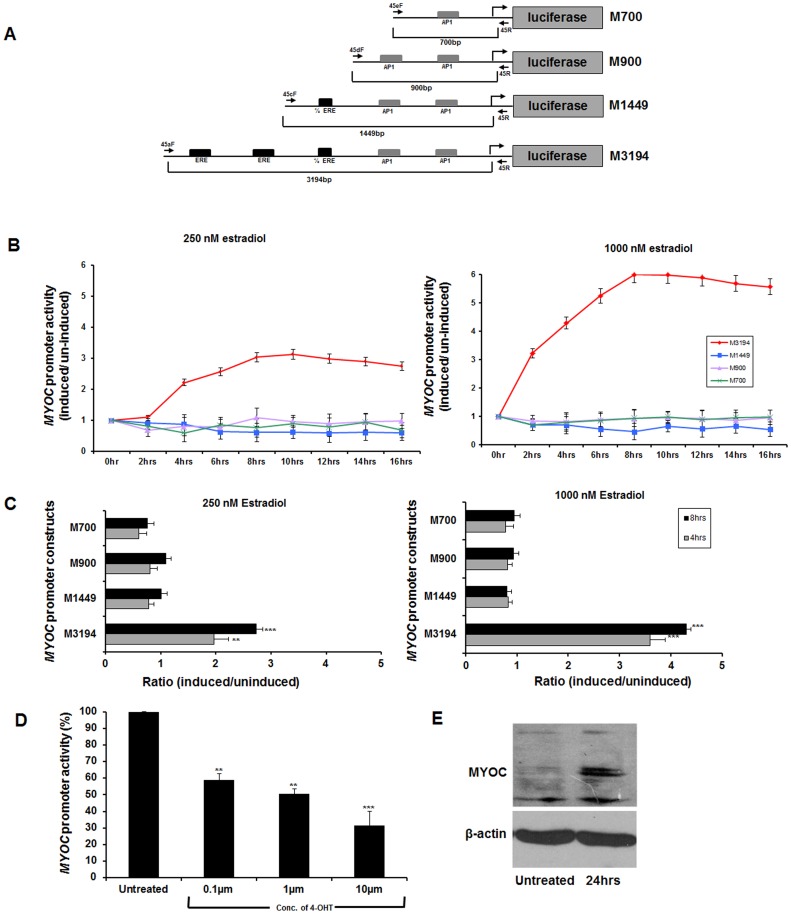
Functional evaluation of putative EREs in *MYOC* promoter. *A*: Serial constructs of *MYOC*-promoter region containing ERE and AP1 sites cloned in promoter less PGL3 basic vector. Black solid arrows indicate the forward and reverse primers used to amplify the inserts for subcloning. Also, the alphanumeric nomenclature of the constructs corresponds to the first initial of *myocilin* (M) followed by the size of the insert in base pairs. ***B*** **:** Luciferase activity in extracts from RPE cells transfected with the clones containing *MYOC* constructs and treated with 17β estradiol (250 nM or 1000 nM). ***C*** **:** Ratio of luciferase activity in cell extracts between induced and uninduced RPE cells for all 4 serial constructs upon dose (250 nM and 1000 nM) and time (4 hrs & 8 hrs) dependent treatment of 17β estradiol. The time points were taken based on the previous experiment in *Panel B*. ***D*** **:** The M3194 construct was transfected in RPE cells and subjected to increasing amount of 4-hydroxy tamoxifen (4-OHT; 17β estradiol competitor) treatment followed by luciferase assay. A gradual decrease in *MYOC* promoter activity was observed with increasing amount of 4-OHT. ***E*** **:** Significant upregulation of endogenous myocilin with 17β estradiol treatment in HTM cell. (**p-value<0.001, ***p-value<0.0001). Three independent replicates were performed for all the experiments described here.

To provide evidence for 17β estradiol mediated upregulation of *MYOC*, it was important to examine the responsiveness of EREs in the *MYOC* promoter in the presence of estradiol. To examine this possibility, deletion constructs (M700, M900, M1449 & M3194 in Figure 3A) of *MYOC* promoter region containing ERE & AP1 sites were generated and cloned in a promoter-less pGL3 basic vector containing luciferase as the reporter gene. To test the functionality of the EREs and AP1 cis-elements, the deletion clones were transfected into human RPE cells, followed by a dose (250 nM and 1000 nM) and time (0–16 hrs) dependent treatment of 17β estradiol, 40 hours post transfection. Based on experiments shown in Figure 3B, two different time points (4 hrs and 8 hrs) were selected for further downstream analysis to determine the ratio of induced and uninduced cells with different dosages of 17β estradiol (Figure 3C). The largest construct (M3194) containing all 3 EREs was observed to induce maximum luciferase activity proportional to dose and duration of estradiol treatment. However, other clones either lacking (M700 & M900) or containing half-ERE (M1449) were unresponsive to 17β estradiol treatment (Figure 3B & 3C).

### Inhibition of *MYOC* Promoter Activity by 17β-estradiol Competitor

To further confirm that the putative EREs in the *MYOC* promoter are active, we treated the cells with Estrogen Receptor (ER) competitor 4-hydroxy tamoxifen (4-OHT). RPE cells were transfected with the largest *MYOC* promoter construct containing all the EREs (M3194) followed by treatment with increasing concentration of 4-OHT. It was found that with increasing concentration of 4-OHT, the basal promoter activity reduced. This observation provided further support that the putative EREs of the *MYOC* promoter are functionally active (Figure 3D).

### 17β Estradiol Elevates the Level of Endogenous Myocilin in Human TM Cells

To further validate the functionality of the putative EREs in *MYOC* promoter, HTM cells were treated with 1000nM of 17β estradiol for 24 hrs. A subsequent increase in the level of endogenous myocilin was observed post treatment (Figure 3E). Thus, this further substantiates the activity of the EREs on *MYOC* promoter and that the increased concentration of 17β estradiol can upregulate *MYOC* in HTM cells.

### 17β Estradiol is Responsible for Nuclear Localization of ERα in HTM and RPE Cells

**Figure 4:**
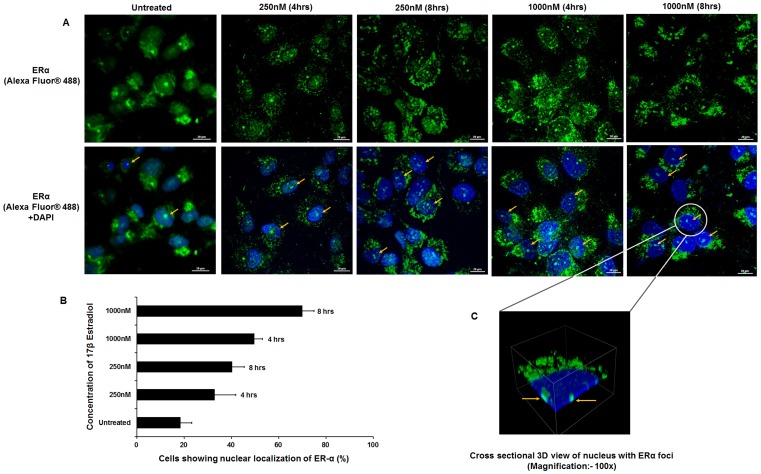
Nuclear localization of ERα on 17β estradiol treatment in HTM cells. *A*: Confocal images of HTM cells upon dose (250 mM & 1000 mM) and time (4 hr and 8 hr) dependent treatment with 17β estradiol. Cells were stained with human specific ERα-antibody followed by Alexa Fluor® 488 labeled anti-rabbit secondary antibody (*Upper panel*). For all conditions, corresponding superimposed image with DAPI are given (*Lower panel*). Arrows point to the cells where nuclear localization of ERα was observed. ***B*** **:** Histogram showing the percentage of HTM cells with ERα localized in the nucleus upon treatment with 17β estradiol in a dose and time dependent manner. Each experiment was done in triplicate. ***C*** **:** Cross sectional 3D view of nuclear localization of ERα in HTM cell is shown [Scale bar: 20 µm].

**Figure 5:**
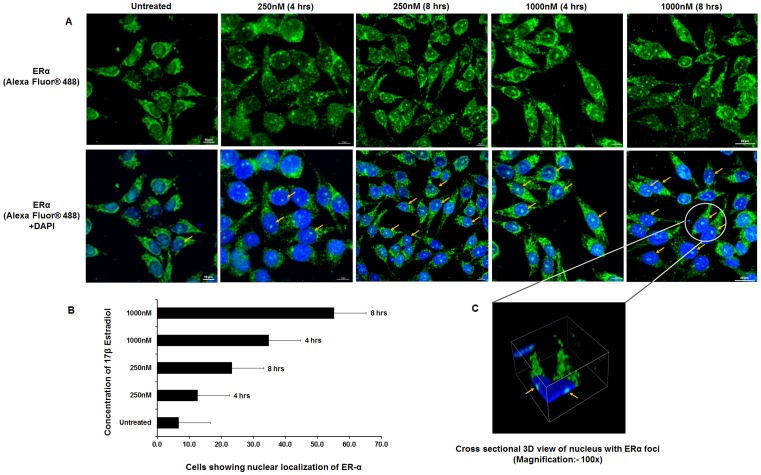
Nuclear localization of ERα upon 17β estradiol treatment in human RPE cells. *A*: Confocal images of human RPE cells upon dose (250 mM & 1000 mM) and time (4 hr and 8 hr) dependent treatment with 17β estradiol. Cells were stained with human specific ERα-antibody followed by Alexa Fluor® 488 labeled anti-rabbit secondary antibody (*Upper panel*). For all conditions, corresponding superimposed image with DAPI are given (*Lower panel*). Arrows point to the cells where nuclear localization of ERα was observed. ***B*** **:** Histogram showing the percentage of RPE cells with ERα localized in the nucleus upon treatment with 17β estradiol in a dose and time dependent manner. Each experiment was done in triplicate. ***C*** **:** Cross sectional 3D view of nuclear localization of ERα in RPE cell is shown [Scale bar: 10 µm].

To examine the presence of ER**α** in HTM and RPE cells and its nuclear translocation on steroid treatment, the cells were treated with 17β estradiol with similar dosage(250 nM and 1000 nM) in a time dependent manner (4 hrs and 8 hrs). Then the cells were treated with human specific ER**α** antibody raised in rabbit followed by treatment with Alexa Fluor® 488 conjugated anti-rabbit secondary antibody. Confocal images of the HTM and RPE cells revealed that nuclear localization of ER**α** was maximum after 8hours at both 250 nM and 1000 nM concentrations of 17β estradiol (Figure 4 and 5).

### ERα Binds to Putative EREs in *MYOC* Promoter

**Figure 6:**
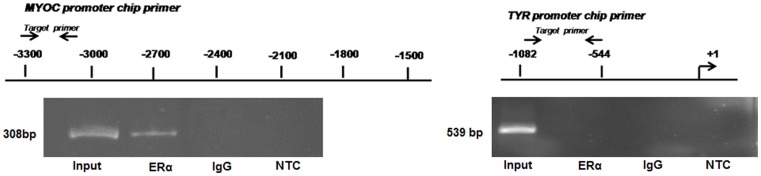
Direct binding of ER-α- 17β estradiol complex to EREs in *MYOC* promoter. ChIP (Chromatin-immunoprecipitation) assay results showing the presence of specific 308 bp band for *myocilin* promoter in the sample immunoprecipitated with the anti-estrogen receptor antibody. No specific band was observed for *Tyrosinase* gene promoter devoid of any EREs. In both the cases no amplification was observed on immunoprecipitation with immunoglobulin (IgG). Three independent replicates were performed for this experiment. NTC: No Template Control.

Since increased concentration of 17β estradiol in HTM cells can upregulate endogenous myocilin and also cause nuclear localization of ERα, we intended to test the direct binding of ERs to the *MYOC* promoter element by chromatin immunoprecipitation (ChIP) assay. In HTM cells, treated with 1000 nM of 17β-estradiol for 48 hrs, ChIP was done using anti-ERα antibody and the precipitated DNA was PCR amplified with primers specific to a 308 bp region of the *MYOC* promoter (Figure 6). Positive amplification of the specific region on *MYOC* promoter was observed in HTM cells treated with 17β-estradiol indicating that ERα specifically binds to the EREs in the *MYOC* promoter and transactivates it. In order to further evaluate the specificity of the ChIP assay, a second PCR amplification was done with the same DNA sample immumoprecipitated with ERα antibody using primers specific to the *Tyrosinase (TYR)* promoter which is devoid of any putative ERE; absence of any PCR product strongly suggests the specific binding of the ERα to the EREs in the *MYOC* promoter (Figure 6).

### CYP1B1 Mutant Proteins Show Reduced Enzymatic Activity

**Figure 7:**
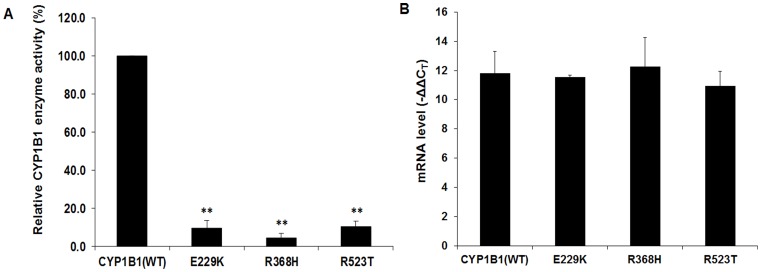
CYP1B1 mutants have lower 17β estradiol metabolizing activity compared to wild type protein. *A*: The enzyme activity of the mutant proteins was expressed as percentage of the activity retained as compared to the native (wild type) enzyme. Mutant constructs of CYP1B1 (i.e. E229K, R368H and R523T) showed <10% of 17β estradiol metabolizing activity (**p-value<0.001). ***B*** **:** Histogram showing the expression level of the transfected wild type and mutant constructs of *CYP1B1* in RPE cells as detected by RT-PCR. Three independent replicates were performed for this experiment.

The enzymatic activity of normal and mutant CYP1B1 was analyzed to explore whether the mutant CYP1B1 clones (E229K, R368H and R532T) lack 17β estradiol metabolizing activity, leading to accumulation of higher level of the steroid in cells. Among the mutants, E229K has been reported as a hypomorphic *CYP1B1* variant [16]. The R523T variant has been reported as a cause of JOAG in an East Indian family [14] and R368H is a predominant mutation in *CYP1B1* in Indian PCG as well as POAG patients [20], [21]. The level of activity of transfected CYP1B1 was measured using P450-Glo assay kit with appropriate controls in each step which includes (i) equal seeding density, (ii) normalization of endogenous CYP1B1, activity, and (iii) transfection efficiency. For each mutant or wild type CYP1B1 enzyme activity was normalized with the transfection efficiency as determined by the mRNA level by real-time PCR (Figure 7B). All three mutants showed <10% 17β metabolizing activity as compared to the wild type CYP1B1 (Figure 7A).

### Elevation of Endogenous Myocilin Level in Mutant CYP1B1 Background

**Figure 8:**
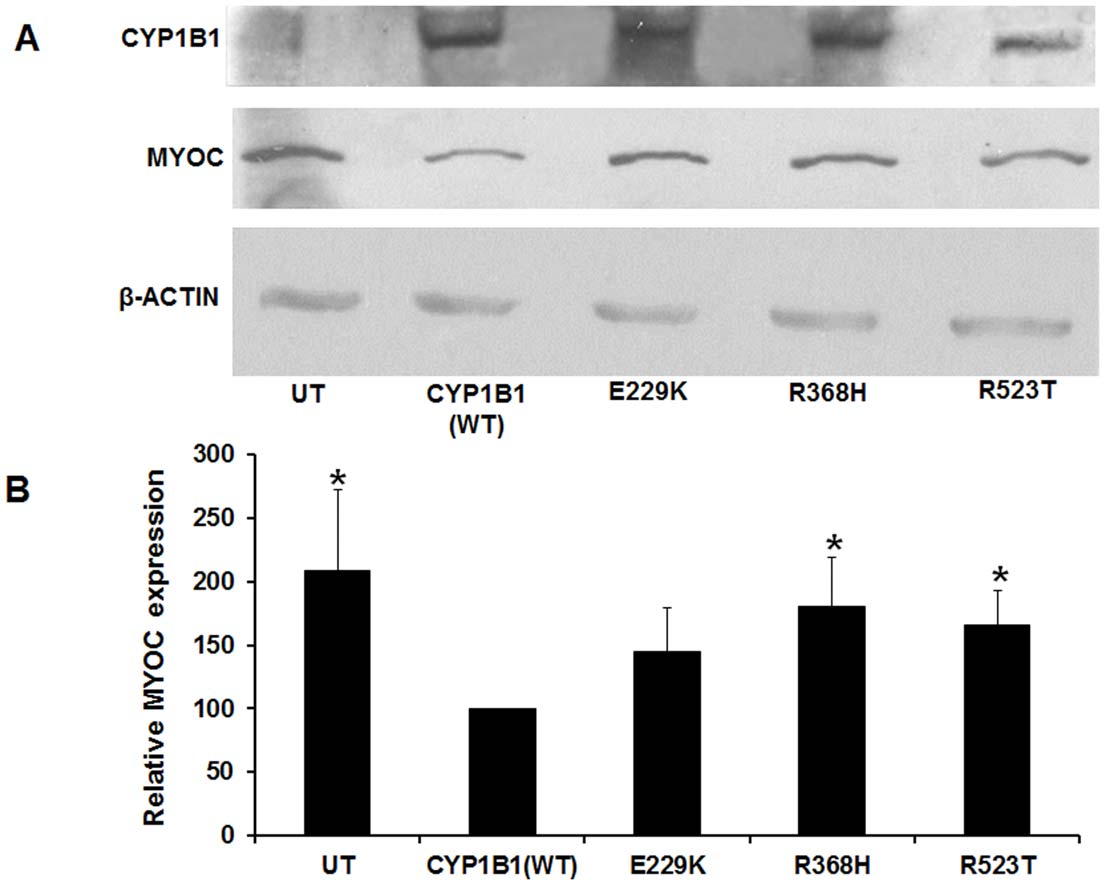
CYP1B1 mutants cause upregulation of MYOC in HTM cells. *A*: *Increased MYOC expression with mutant CYP1B1*. Western blot analysis of mutant CYP1B1 and myocilin showed increased expression of MYOC in the presence of mutant CYP1B1 clones with reduced (<10%) 17β estradiol metabolizing activity. ***B*** **:** *Quantitative analysis of MYOC expression*. The histogram shows levels of expression of endogenous MYOC in HTM cells transfected with wild type and mutant CYP1B1 clones. All the three mutants of CYP1B1 (i.e. E229K, R368H and R523T) considerably over-expressed MYOC compared to the normal CYP1B1. The R368H and R523T showed statistically significant over expression of myocilin with a p-value of 0.023 and 0.014, respectively. However, the effect of E229K mutant was not found to be statistically significant. This experiment was repeated three times [*p-value- <0.05].

To delineate the effect of CYP1B1 mutants on the expression of myocilin, the level of endogenous myocilin in HTM cell line was analyzed in cells transfected with mutant CYP1B1 constructs. The cells were harvested 36 hrs after transfection with normal and mutant CYP1B1 constructs (E229K, R523T and R368H). Myocilin expression in the background of R368H and R523T mutants was found to be significantly higher leading to 180% (p value: 0.023) and 165% (p value: 0.014) expression of MYOC, respectively, compared to normal CYP1B1. The E229K mutant of CYP1B1, though over expressed myocilin by 144% as compared to wild type CYP1B1, the expression was not found to be statistically significant (p value: 0.086) (Figure 8). Thus, in cells expressing mutant CYP1B1 consistent overexpression of MYOC was observed.

## Discussion

To date multiple genetic evidences of involvement of *CYP1B1* in POAG have been reported [12]–[14], [22], [23]. From these reports, it appears that *CYP1B1* has a larger role to play in glaucoma pathogenesis, which includes causation of PCG, acting as a modifier for POAG and on rare occasions, being the primary cause of JOAG. In addition, digenic inheritance in POAG suggested genetic correlation between *MYOC* and *CYP1B1* that requires to be understood at the functional level. In our present study, we deciphered that CYP1B1 plays a role in transcriptional activation of *MYOC,* by regulating the level of 17β estradiol, the substrate of *CYP1B1*.

It was observed that 17β estradiol is present in ocular cells (HTM and RPE), which was further supported by the expression of the enzymes required to synthesize estrogen from cholesterol in these cells. Previous studies have demonstrated presence of estradiol synthesizing enzymes in human ocular surface and adnexal tissues (lacrimal and meibomian glands, corneal and conjunctival epithelium, ciliary epithelium etc.) [24], [25]. Expression of sex steroid hormone receptors were also found in multiple ocular tissues (cornea, conjunctiva, ciliary body, retina, RPE etc.) in different animals [26]. These observations together point that potential local sex steroid biosynthetic and effector mechanisms are available in the eye.

To support our hypothesis, we established that the EREs in the *MYOC* promoter [27] could induce its transcription in the presence of 17β estradiol. Also by the introduction of 17β estradiol competitor it was found that the basal promoter activity of the *MYOC* promoter is inhibited, which further proves that the EREs of the *MYOC* promoter are active. In addition, a twofold higher expression of endogenous myocilin was observed in HTM cell line upon treatment with 17β estradiol. This was further supported by observed nuclear localization of estrogen receptor-alpha on17β estradiol induction. This transactivation was eventually brought about by binding of estrogen receptor and 17β estradiol complex on the ERE elements of the *MYOC* promoter, as evidenced from our ChIP experiment results.

It is well known that CYP1B1 plays an important role in steroid metabolism (4-hydroxylation of 17β estradiol). Induction of *MYOC* by dexamethasone, which is a corticosteroid by nature [28] and association of early menopause with POAG [29], point towards the fact that regulation of 17β estradiol by CYP1B1 is likely to have a role in POAG. In fact, we detected the over-expression of endogenous myocilin in HTM cell line in the presence of mutated CYP1B1. The three *CYP1B1* mutants (E229K, R368H and R523T), earlier detected in East Indian POAG patients [14], were selected for the present study and were found to have <10% of relative enzymatic activity compared to the wild type. Among these three mutations, R368H is one of the predominant mutations in India in both PCG and POAG and R523T is a novel mutation found to cause JOAG in autosomal recessive mode of inheritance. Interestingly, for both the mutations significant increase in myocilin expression was found. Although E229K did not elicit significant overexpression of myocilin, it is possible that, similar to other multifactorial diseases, additional underlying factor(s) might precipitate the disease. E229K has been previously characterized as a hypomorphic allele [16]. Nevertheless, the activity for the variant (E229K) has been reported to be 26–40% [15], [16] while we observed it to be <10%. The difference in *in-vitro* assessment of the enzyme activity may be due to the usage of different cells (RPE, HEK-293 or yeast cells), assay method and method for normalization (protein vs mRNA). In addition, relatively lower stability of E229K [16] could further complicate the precise assessment of the enzyme activity. Also, it is predicted to act as a risk factor for both PCG and POAG [14], [20], [21]. However, this variant has not yet been found in homozygous state in PCG patients [16]. Even when found in compound heterozygous state with another mutation, E229K was not found to be fully penetrant [23], [30]. Interestingly, we found that this variant does not significantly overexpress myocilin.

*CYP1B1* mutation has also been found in sporadic POAG patients and on rare occasions, can cause JOAG in autosomal recessive mode of inheritance [14]. *MYOC* mutation when present along with *CYP1B1* mutation is reported to expedite the disease condition [31]. Also, mutated myocilin has been reported to form protein aggregates in the cell, in cytoplasm as well as in endoplasmic reticulum which ultimately results in cell death [32]. Therefore, over expression of mutated myocilin as a result of mutant CYP1B1 would be expected to hasten up the cell death. Over expression of wild type myocilin is also believed to be involved in glaucoma pathogenesis. It is believed that in case of steroid induced glaucoma, over expression myocilin is one of the triggering factors for glaucoma causation; however, the notion is still controversial [33]. Over expression of myocilin has been shown to compromise the adhesive property of the cultured HTM cells through activation of cAMP/PKA and inhibition of Rho kinase [34]. Human myocilin over expression in the eyes of *Drosophila melanogaster* results in distortion of ommatidia (the ‘eye’ of Drosophila) which is accompanied by fluid discharge [35] and also activates the UPR (Unfolded protein response) [36]. On the contrary, BAC mediated myocilin overexpression in mouse does not produce any glaucoma phenotype [37]. This is not rare that mutations in homologues genes in mouse do not produce similar phenotype observed in human, which also varies depending on the mouse strain used in the specific study. Intravitreal administration of adenoviral vector with Y437H myocilin mutation in four different mouse strain (A/J, BALB/cJ, C57BL/6J, and C3H/HeJ) elevated the IOP level in three (BALB/cJ, C57BL/6J, and A/J) and considerable damage to the optic nerve was found in only one strain (A/J) [38].

**Figure 9:**
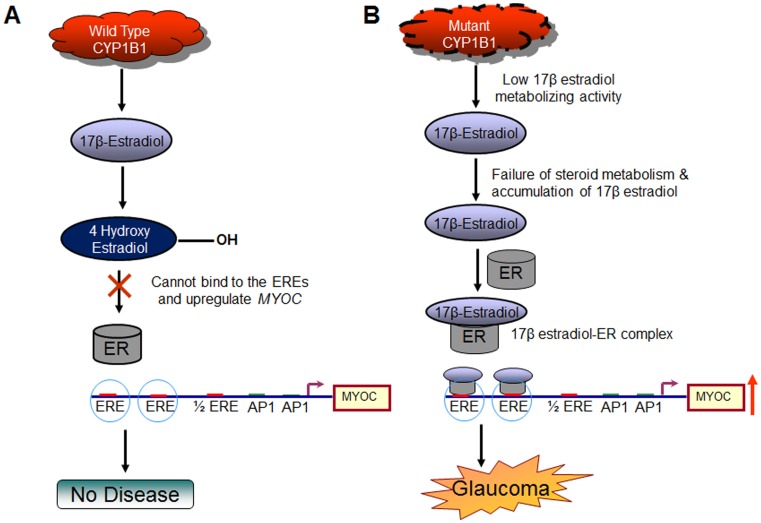
Schematic diagram showing potential influence of CYP1B1 mutants on MYOC expression. In ***Panel A*** fully functional wild-type CYP1B1 metabolizes 17β-Estradiol; thus limiting the steroid to form the hormone-receptor complex (17β-Estradiol-ER) whereas in ***Panel B*** restricted CYP1B1 enzymatic activity results in higher levels of the steroid available for formation of 17β-Estradiol-ER complex which in turn leads to *MYOC* upregulation through estrogen response elements (EREs) in *MYOC* promoter. The latter condition might have a potential implication in glaucoma pathogenesis.

*CYP1B1* represents the first example where mutation in a member of the cytochrome P450 superfamily results in a primary developmental defect in terms of PCG [11]. It has also been found to be associated with head and neck squamous cell carcinoma and breast cancer [39], [40]. It has been speculated earlier that CYP1B1 participates in the metabolism of an as-yet-unknown biologically active molecule that is a participant in eye development [11]. Vincent *et al* (2001) [12] suggested that *MYOC* and *CYP1B1* possibly interact through a common pathway and proposed that myocilin function could potentially be influenced by mutation in CYP1B1. Also, polymorphisms in *CYP1B1* are known to be associated with its functional differences in estrogen hydroxylation activity [41]. Here we tried to elucidate a possible underlying mechanism where regulation of 17β estradiol by CYP1B1 plays a key role in the cascade of events that may lead to glaucoma (Figure 9). However, further *in-vivo* studies on animal models are necessary to provide additional supporting evidence for CYP1B1 mediated MYOC upregulation as a molecular basis for glaucoma pathogenesis.

## Methods

### Mammalian Cell Culture

The human retinal pigment epithelium cell line RPE8319 (kind gift from Dr. Frans Cremers, Univ. Medical Center, Nijmegen, The Netherlands), HEK293, and Human Trabecular Meshwork (HTM) cell line (originally developed by Polansky *et al* and kindly gifted by Dr. Michael Walter, University of Alberta, Edmonton, Canada) were maintained in DMEM (Dulbecco’s modified Eagle Medium, GIBCO BRL) at pH 7.4 supplemented with 10% fetal bovine serum (GIBCO BRL) containing penicillin/streptomycin/gentamycin in the presence of 5% CO_2_ at 37°C. While RPE and HEK293 cell lines were maintained with high glucose concentration (4 g/L), HTM cell line was maintained in media containing lower glucose concentration (1 g/L).The identity of the HTM cell line was verified by upregulation of endogenous myocilin by dexamethasone treatment (Figure S1). Transient transfections of human RPE and HTM cell lines were performed with the Lipofectamine 2000 system according to the manufacturer’s instructions (Invitrogen Inc., USA).

### Immunocytochemistry

For the immunofluorescence assay, HTM and RPE cells (approximately 3×10^5^ cells) were cultured on coverslips in a 35 mm culture dish for 14 hrs. The cells were rinsed twice with cold PBS and fixed with cold acetone:methanol (1∶1) fixing solution for 5 mins at −20°C. After fixing, the cells were washed with PBS 3 to 4 times. Only in case of ER-α, cells were permealized using 0.1% Triton-X (Sigma-Aldrich, USA) at room temperature for 3 mins. The cells were then incubated in 5% BSA (Bangalore Genei, India) in PBS for 1hour at room temperature. This was followed by overnight incubation with either human ER-α specific primary antibody (1∶100 dilution) raised in rabbit (Sigma Biologicals, Germany) or human 17β estradiol specific primary antibody (1∶50 dilution) raised in mouse (Geneway, USA) at 4°C. On the following day, the excess primary antibody was washed off with PBS thrice and the cells were incubated with Alexa Fluor® 488 anti rabbit (1∶200 dilutions) (Invitrogen, USA) or FITC conjugated anti mouse secondary antibody (1∶200 dilutions) (Bangalore Genei, India) for 2hours at room temperature. The cells were again washed with PBS thrice and the cover slips were mounted on glass slides with Prolong Gold Antifade reagent with DAPI (Invitrogen, USA). The confocal imaging was done using A1R confocal microscope (Nikon, Japan).

To quantify ERα nuclear localization 3 to 4 fields were randomly chosen and percentage of cells showing ERα foci in the nucleus was determined for each field (for both RPE and HTM cells). The experiment was done in triplicate. In each case nuclear localization of the ER-α was confirmed by 3D imaging of the cells.

### Detection of 17β Estradiol in HTM, RPE and Culture Media by ELISA

Assessment of 17β estradiol in cells and cell culture media was done using estradiol bioassay kit (Stressgene, USA) with a modified protocol. The HTM and RPE cells were grown to confluency in a 6 cm culture dish in a media containing charcoal treated FBS for several passages. The confluent HTM and RPE cells were treated with 500 µl of NP40 lysis buffer and the lysate was collected in a tube. Estradiol was extracted from cell lysate with diethyl-ether. Estradiol was also extracted from the “virgin” culture media (not used in cell culture procedure) with charcoal treated FBS to estimate the level of estradiol in the media. After extraction the ether was evaporated under nitrogen and the extracted estradiol was re-suspended in assay buffer provided with the kit.

The kit is provided with a 96 well ELISA plate pre-coated with goat anti rabbit secondary antibody. The external antigen i.e 17β estradiol has to compete with an ALP (alkaline phosphatase) conjugated 17β estradiol competitor provided with the kit for binding with estradiol specific primary antibody raised in rabbit. So, as the amount of the external antigen increases the ALP signal is decreased. The amount of external antigen was calculated from the standard graph prepared with known amount of 17β estradiol. This experiment was repeated three times.

### Construction of *MYOC*-promoter Clones in PGL3 Basic Vector

Serial fragments containing AP1 and EREs in the promoter region of *MYOC* were amplified from genomic DNA using the respective oligonucleotide pairs (Table S1). All the oligonucleotides used for this purpose contained *Xho*I and *Hind*III restriction site at their 5′- and 3′ ends respectively. The fragments were then cloned either directly or via TA vector (pCR2.1, Invitrogen, USA) mediated cloning into PGL3 basic vector (Promega, USA) after digestion with *Xho*I and *Hind*III (New England Biolabs, USA) using T4 DNA ligase (Invitrogen, USA). Recombinant plasmids were isolated using QIAGEN plasmid midi kit (Qiagen, Germany) from transformed *E.coli* cells in ampicillin mediated selection condition. These constructs were first checked on 0.8–1.0% agarose gel after digestion with above mentioned enzymes and then sequenced using respective amplification primers (Table S1) on one end and PGL3 specific primer PGLR (5′-CTTTATGTTTTTGGCGTCTTCCA-3′) from the other end.

### Assay for Luciferase Activity Following Treatment with 17β Estradiol and 4-hydroxy-tamoxifen (4-OHT)

Human RPE cells were (approximately 1×10^6^ cells/per well) seeded on 6-well plate and left overnight (∼14 hrs). Cells were transiently transfected with empty pGL3 vector as control and *MYOC* promoter constructs. For consistency each transfection was carried out with 2 µg of either *MYOC* promoter constructs or pGL3 control vector from a single plasmid preparation. After 36 hours of transfection RPE cells were subjected to dose and time dependent treatment of 17β estradiol followed by luciferase assay. The luciferase activity was normalized with protein concentration and the ratio between estradiol treated and untreated cells were taken. This experiment was repeated three times and statistical significance was calculated using student’s t-test.

For examining the inhibition of myocilin promoter activity by estrogen receptor competitor, human RPE cells (approximately 1×10^6^ cells/per well) were seeded on 6-well plate and cultured overnight (∼14 hrs). The cells were then treated with 0.1 µM, 1 µM and 10 µM of 4-OHT (Sigma, USA) for 8 hrs, post 36 hrs of transfection with M3194 (2µg/per well); the largest construct containing all the EREs followed by luciferase assay. For consistency replicate experiments were carried out with plasmids from single preparation. Here also the luciferase activity was normalized with protein concentration and the fold differences between 4-OHT treated and untreated cells were taken. For each dose luciferase activity was measured for three independent experiments and statistical significance was calculated using student’s t-test.

The luciferase activity was determined as light units using the Luciferase Assay Kit (Promega, Madison, USA). For the assay, the cells were washed with phosphate buffered saline and lysed in the luciferase cell culture lysis buffer provided with the Luciferase Assay Kit. Twenty five micro-liter of cell lysate was then added to 25 µl of luciferase assay substrate (Luciferase Assay Kit) and the luminescence was measured as light units in a Monolight 2010 luminometer (Analytical Luminescence, San Diego, CA). The protein concentration in each lysate was determined with a protein assay kit (Bio-Rad, USA) and used to normalize the luciferase activity.

### Chromatin Immunoprecipitation (ChIP) Assay

For ChIP assay HTM cells were treated with 17β estradiol for 48 hrs. Then the cells were processed for ChIP reaction with IMGENEX quick ChIP kit (IMGENEX, USA) according to the manufacturer’s protocol using estrogen receptor specific antibody (Sigma, USA) or IgG (negative control) (primer lists are available in Table S2).

### Site Directed Mutagenesis

A CYP1B1 cDNA construct in the pcDNA3 mammalian expression vector (kindly gifted by Dr. Thomas. H. Friedberg University of Dundee, UK) was used to generate mutant CYP1B1 clones: c.1057 G>A (E229K), c.1475 G>A (R368H) and c.1940 G>C (R523T). Site-directed mutagenesis and subsequent transformation of the mutant clones were performed using the QuikChange XL Site-Directed Mutagenesis Kit (Stratagene, La Jolla, CA, USA) according to the manufacturer’s protocol with specific primers (Table S3 Table S4). Plasmid isolation was done for all the three variants c.1057 G>A (E229K), c.1475 G>A (R368H) and c.1940 G>C (R523T) cloned in the pcDNA3 vector, using a QIAGEN plasmid mini kit (Qiagen, Germany) according to the manufacturer’s protocol. The targeted changes in the recombinant clones generated were confirmed by sequencing.

### Determination of Wild Type and Mutant CYP1B1 Enzyme Activity

RPE cells (approximately 3×10^5^ cells per well) were grown on 12 well culture plates. After 24 hrs, cells were transfected with 2 µg wild type and mutant CYP1B1 (pE229K, pR368H and pR523T). Forty hours post transfection CYP1B1 activity was measured using the CYP450-GLO™ Assay kit (catalog number V8762; Promega, Madison, WI) with a modified protocol. The media was changed and the luciferin-CEE substrate (0.1 mM final concentration) was added to each well with 500 µl of media. After 3½hrs incubation most of the luciferin-CEE was converted to luciferin and secreted out in the media. The medium was collected from each well and processed according to the manufacturer’s protocol. The final amount of luciferin was determined using luminometer.

For each observation total media (500 µl) was taken out and 3 aliquots of 50µl of media for each mutant as well as the wild type construct were used for final reading from luminometer. The relative luminescence unit (RLU) obtained was normalized with respective mRNA levels of the wild type and mutant CYP1B1, as determined by Real-Time PCR, to nullify the difference in transfection efficiency. The enzyme activity of the mutant proteins were expressed as percentage of activity retained as compared to wild type protein. For each mutant three independent observations were made and statistical significance was calculated using Student’s t-test.

### Preparation of cDNA from RNA (RT) and PCR

To determine the RNA levels of 17β estradiol synthesizing enzymes and the wild type and mutant CYP1B1, cells were collected from respective wells after determination of the luciferase activity from the media. Total RNA was extracted from each by using TriZol™ (Invitrogen, USA) as per the manufacturer’s protocol. Preparation of cDNA was done using SUPERSCRIPT™ First –Strand Synthesis System (Invitrogen, USA) for RT-PCR at 42°C for 50 min following the manufacturer’s instructions.

For PCR about 2 µl of cDNA was amplified in a 30 µl reaction volume containing 10 mM Tris-HCl, pH 8.3; 50 mM KCl, 2.5 mM or 2.0 mM MgCl_2_ as required, 0.26mM of each dNTPs and 25 pmol of each primer and 0.5U Taq DNA polymerase (GIBCO-BRL, Gaithersburg, MD). To determine the expression of the enzymes required for estrogen synthesis from cholesterol, cDNAs of HTM and RPE cells were amplified with primer pairs (Table S4) from coding sequence but encompassing an intron such that amplified cDNA product could be distinguished from the gDNA by the known difference in sizes of the amplicons.

### Quantitative Real Time-PCR for Normal and Mutant CYP1B1

The mRNA expression of normal and mutant (c.1057 G>A, c.1475 G>A and c.1940 G>C) variants of *CYP1B1* was determined by Real-Time PCR in the 7500 Real Time PCR System (Applied Biosystems, Foster City, CA) using SYBR Green Jumpstart Taq Ready Mix (Sigma, St.Louis, MO). The following primers were used: CYP1B1 F 5′-GGAGAACGTACCGGCCACTATC-3′ and R 5′-CTTGGGTTTAATGGTTAGACC-3′ and human β-actin F 5′-TGACGGGGTCACCCACACTGTGCCCATCTA- 3′ and R 5′-CTAGAAGCATTGCGGTGGACGATGGAGGG- 3′. The threshold cycle C_T_ of duplicate samples was determined using 7500 System SDS software (Applied Biosystems, Foster City, CA). The levels of both normal and mutant *CYP1B1* were normalized to β-actin levels by calculating the ΔC_T_ value, which is the C_T_ (threshold cycle) of the housekeeping gene (β-actin) subtracted from the C_T_ of the target gene (*CYP1B1*). As CYP1B1 mRNA is expressed in RPE cells, we further normalized the data by calculating the ΔΔC_T_ value which is a subtraction of ΔC_T_ value of untransfected cells from each ΔC_T_ value of both cells transfected with normal and mutant *CYP1B1*. All calculations were done according to the published paper in 2001 by Giulietti et al [42].

### Protein Electrophoresis and Western Blotting

Human trabecular meshwork (HTM) cells were grown on 6 well culture plates. After 24 hrs, cells were transfected with 4 µg of wild type and mutant CYP1B1 constructs. After transfection, the cells were treated with monensin sodium salt (3.2 µg/ml of media) for 36 hrs. Thirty six hrs post transfection cells were lysed using NP-40 lysis buffer (150 mM Tris-Cl, 50 mM EDTA, pH. 8.0, 1%NP40) supplemented with protease inhibitor cocktail (1ul/106 cell; Sigma, St.Louis, MO) and was sonicated in a water bath for 5 mins. Total protein was estimated using Bradford assay, which is necessary for equal loading of the lysates. Fifty microgram of each protein sample were resolved in 10% SDS-polyacrylamide (MiniPROTEAN III; BioRad, Herucles, CA) and transferred onto a PVDF membrane (Hybond-P; GE Healthcare, Bedford, UK) by electroblotting using the ECL semi-dry transfer unit (Amersham Biociences, USA). Membranes were then blocked in 5% BSA in TBS for 3 hrs at room temperature and incubated with respective primary antibody [anti-CYP1B1 polyclonal antibody (1∶1000) (Abcam, UK), anti-MYOC polyclonal antibody (1∶250) (SantaCruz, USA) and Anti-β actin (1∶2000) antibody (Sigma-Aldrich, USA)] overnight at 4°C. Βeta- actin was used as the loading control. The membranes were washed thrice with TBST [25 mM Tris-HCl (pH 7.5), 150 mM NaCl, 0.05% tween-20] at 10 mins interval followed by incubation with appropriate secondary antibody conjugated with HRP [Anti-rabbit (1∶60,000) and anti-mouse (1∶2000)] (Bangalore Genei, India) for 2 hrs at room temperature. The secondary antibody was washed thrice with TBST followed by two washes with TBS. The ECL-western blotting detection kit (Pierce, USA) was used for chemiluminiscent detection. Densitometry for protein band quantitation was performed on scanned films using Image J software on triplicated independent experiments. The results were compared using Student’s t-test.

## Supporting Information

Figure S1 **Upregulation of myocilin upon dexamethasone treatment.** A considerable overexpression of myocilin in HTM cell line is observed upon treatment with 100 mM dexamethasone for 5 days
(DOC)

Table S1 **Primer sequences and PCR conditions for serial amplification of promoter regions in** ***MYOC.***
(DOCX)

Table S2 **Primers used for ChIP assay.**
(DOCX)

Table S3 **Primers for site directed mutagenesis of CYP1B1 constructs.**
(DOCX)

Table S4 **Primers used for RT-PCR to amplify genes in estrogen synthesis pathway.**
(DOCX)

## References

[1] Libby RT, Gould DB, Anderson MG, John SW (2005) Complex genetics of glaucoma susceptibility. Annu Rev Genomics Hum Genet 6: 15–44. 16124852 10.1146/annurev.genom.6.080604.162209

[2] Pascolini D, Mariotti SP (2012) Global estimates of visual impairment: 2010. Br J Ophthalmol 96: 614–618. 22133988 10.1136/bjophthalmol-2011-300539

[3] Ray K, Mookherjee S (2009) Molecular complexity of primary open angle glaucoma: current concepts. J Genet 88: 451–467. 20090207 10.1007/s12041-009-0065-3

[4] Gong G, Kosoko-Lasaki S, Haynatzki G, Lynch HT, Lynch JA, et al (2007) Inherited, familial and sporadic primary open-angle glaucoma. J Natl Med Assoc 99: 559–563. 17534014 PMC2576058

[5] Stone EM, Fingert JH, Alward WL, Nguyen TD, Polansky JR, et al (1997) Identification of a gene that causes primary open angle glaucoma. Science 275: 668–670. 9005853 10.1126/science.275.5300.668

[6] Rezaie T, Child A, Hitchings R, Brice G, Miller L, et al (2002) Adult-onset primary open-angle glaucoma caused by mutations in optineurin. Science 295: 1077–1079. 11834836 10.1126/science.1066901

[7] Monemi S, Spaeth G, DaSilva A, Popinchalk S, Ilitchev E, et al (2005) Identification of a novel adult-onset primary open-angle glaucoma (POAG) gene on 5q22.1. Hum Mol Genet 14: 725–733. 15677485 10.1093/hmg/ddi068

[8] Pasutto F, Matsumoto T, Mardin CY, Sticht H, Brandstatter JH, et al (2009) Heterozygous NTF4 mutations impairing neurotrophin-4 signaling in patients with primary open-angle glaucoma. Am J Hum Genet 85: 447–456. 19765683 10.1016/j.ajhg.2009.08.016 PMC2756554

[9] Vithana EN, Nongpiur ME, Venkataraman D, Chan SH, Mavinahalli J, et al (2010) Identification of a novel mutation in the NTF4 gene that causes primary open-angle glaucoma in a Chinese population. Mol Vis 16: 1640–1645. 20806036 PMC2927376

[10] Fingert JH, Heon E, Liebmann JM, Yamamoto T, Craig JE, et al (1999) Analysis of myocilin mutations in 1703 glaucoma patients from five different populations. Hum Mol Genet 8: 899–905. 10196380 10.1093/hmg/8.5.899

[11] Stoilov I, Akarsu AN, Sarfarazi M (1997) Identification of three different truncating mutations in cytochrome P4501B1 (CYP1B1) as the principal cause of primary congenital glaucoma (Buphthalmos) in families linked to the GLC3A locus on chromosome 2p21. Hum Mol Genet 6: 641–647. 9097971 10.1093/hmg/6.4.641

[12] Vincent AL, Billingsley G, Buys Y, Levin AV, Priston M, et al (2002) Digenic inheritance of early-onset glaucoma: CYP1B1, a potential modifier gene. Am J Hum Genet 70: 448–460. 11774072 10.1086/338709 PMC384919

[13] Melki R, Colomb E, Lefort N, Brezin AP, Garchon HJ (2004) CYP1B1 mutations in French patients with early-onset primary open-angle glaucoma. J Med Genet 41: 647–651. 15342693 10.1136/jmg.2004.020024 PMC1735887

[14] Acharya M, Mookherjee S, Bhattacharjee A, Bandyopadhyay AK, Daulat Thakur SK, et al (2006) Primary role of CYP1B1 in Indian juvenile-onset POAG patients. Mol Vis 12: 399–404. 16688110

[15] Campos-Mollo E, Lopez-Garrido MP, Blanco-Marchite C, Garcia-Feijoo J, Peralta J, et al (2009) CYP1B1 mutations in Spanish patients with primary congenital glaucoma: phenotypic and functional variability. Mol Vis 15: 417–431. 19234632 PMC2645906

[16] Chavarria-Soley G, Sticht H, Aklillu E, Ingelman-Sundberg M, Pasutto F, et al (2008) Mutations in CYP1B1 cause primary congenital glaucoma by reduction of either activity or abundance of the enzyme. Hum Mutat 29: 1147–1153. 18470941 10.1002/humu.20786

[17] Choudhary D, Jansson I, Sarfarazi M, Schenkman JB (2008) Characterization of the biochemical and structural phenotypes of four CYP1B1 mutations observed in individuals with primary congenital glaucoma. Pharmacogenet Genomics 18: 665–676. 18622259 10.1097/FPC.0b013e3282ff5a36

[18] Lopez-Garrido MP, Blanco-Marchite C, Sanchez-Sanchez F, Lopez-Sanchez E, Chaques-Alepuz V, et al (2010) Functional analysis of CYP1B1 mutations and association of heterozygous hypomorphic alleles with primary open-angle glaucoma. Clin Genet 77: 70–78. 19793111 10.1111/j.1399-0004.2009.01284.x

[19] Pasutto F, Chavarria-Soley G, Mardin CY, Michels-Rautenstrauss K, Ingelman-Sundberg M, et al (2010) Heterozygous loss-of-function variants in CYP1B1 predispose to primary open-angle glaucoma. Invest Ophthalmol Vis Sci 51: 249–254. 19643970 10.1167/iovs.09-3880

[20] Reddy AB, Kaur K, Mandal AK, Panicker SG, Thomas R, et al (2004) Mutation spectrum of the CYP1B1 gene in Indian primary congenital glaucoma patients. Mol Vis 10: 696–702. 15475877

[21] Kumar A, Basavaraj MG, Gupta SK, Qamar I, Ali AM, et al (2007) Role of CYP1B1, MYOC, OPTN, and OPTC genes in adult-onset primary open-angle glaucoma: predominance of CYP1B1 mutations in Indian patients. Mol Vis 13: 667–676. 17563717 PMC2765475

[22] Chakrabarti S, Kaur K, Komatireddy S, Acharya M, Devi KR, et al (2005) Gln48His is the prevalent myocilin mutation in primary open angle and primary congenital glaucoma phenotypes in India. Mol Vis 11: 111–113. 15723004

[23] Lopez-Garrido MP, Sanchez-Sanchez F, Lopez-Martinez F, Aroca-Aguilar JD, Blanco-Marchite C, et al (2006) Heterozygous CYP1B1 gene mutations in Spanish patients with primary open-angle glaucoma. Mol Vis 12: 748–755. 16862072

[24] Coca-Prados M, Ghosh S, Wang Y, Escribano J, Herrala A, et al (2003) Sex steroid hormone metabolism takes place in human ocular cells. J Steroid Biochem Mol Biol 86: 207–216. 14568574 10.1016/j.jsbmb.2003.08.001

[25] Schirra F, Suzuki T, Dickinson DP, Townsend DJ, Gipson IK, et al (2006) Identification of steroidogenic enzyme mRNAs in the human lacrimal gland, meibomian gland, cornea, and conjunctiva. Cornea 25: 438–442. 16670482 10.1097/01.ico.0000183664.80004.44

[26] Wickham LA, Gao J, Toda I, Rocha EM, Ono M, et al (2000) Identification of androgen, estrogen and progesterone receptor mRNAs in the eye. Acta Ophthalmol Scand 78: 146–153. 10794246 10.1034/j.1600-0420.2000.078002146.x

[27] Nguyen TD, Chen P, Huang WD, Chen H, Johnson D, et al (1998) Gene structure and properties of TIGR, an olfactomedin-related glycoprotein cloned from glucocorticoid-induced trabecular meshwork cells. J Biol Chem 273: 6341–6350. 9497363 10.1074/jbc.273.11.6341

[28] Polansky JR, Fauss DJ, Chen P, Chen H, Lutjen-Drecoll E, et al (1997) Cellular pharmacology and molecular biology of the trabecular meshwork inducible glucocorticoid response gene product. Ophthalmologica 211: 126–139. 9176893 10.1159/000310780

[29] Hulsman CA, Westendorp IC, Ramrattan RS, Wolfs RC, Witteman JC, et al (2001) Is open-angle glaucoma associated with early menopause? The Rotterdam Study. Am J Epidemiol 154: 138–144. 11447046 10.1093/aje/154.2.138

[30] Firasat S, Riazuddin SA, Khan SN, Riazuddin S (2008) Novel CYP1B1 mutations in consanguineous Pakistani families with primary congenital glaucoma. Mol Vis 14: 2002–2009. 18989382 PMC2579935

[31] Vincent AL, Billingsley G, Buys Y, Levin AV, Priston M, et al (2002) Digenic inheritance of early-onset glaucoma: CYP1B1, a potential modifier gene. Am J Hum Genet 70: 448–460. 11774072 10.1086/338709 PMC384919

[32] Liu Y, Vollrath D (2004) Reversal of mutant myocilin non-secretion and cell killing: implications for glaucoma. Hum Mol Genet 13: 1193–1204. 15069026 10.1093/hmg/ddh128

[33] Clark AF, Wordinger RJ (2009) The role of steroids in outflow resistance. Exp Eye Res 88: 752–759. 18977348 10.1016/j.exer.2008.10.004

[34] Shen X, Koga T, Park BC, SundarRaj N, Yue BY (2008) Rho GTPase and cAMP/protein kinase A signaling mediates myocilin-induced alterations in cultured human trabecular meshwork cells. J Biol Chem 283: 603–612. 17984096 10.1074/jbc.M708250200 PMC2729092

[35] Borras T, Morozova TV, Heinsohn SL, Lyman RF, Mackay TF, et al (2003) Transcription profiling in Drosophila eyes that overexpress the human glaucoma-associated trabecular meshwork-inducible glucocorticoid response protein/myocilin (TIGR/MYOC). Genetics 163: 637–645. 12618402 10.1093/genetics/163.2.637 PMC1462450

[36] Carbone MA, Ayroles JF, Yamamoto A, Morozova TV, West SA, et al (2009) Overexpression of myocilin in the Drosophila eye activates the unfolded protein response: implications for glaucoma. PLoS ONE 4: e4216. 19148291 10.1371/journal.pone.0004216 PMC2615221

[37] Gould DB, Miceli-Libby L, Savinova OV, Torrado M, Tomarev SI, et al (2004) Genetically increasing Myoc expression supports a necessary pathologic role of abnormal proteins in glaucoma. Mol Cell Biol 24: 9019–9025. 15456875 10.1128/MCB.24.20.9019-9025.2004 PMC517885

[38] McDowell CM, Luan T, Zhang Z, Putliwala T, Wordinger RJ, et al.. (2012) Mutant human myocilin induces strain specific differences in ocular hypertension and optic nerve damage in mice. Exp Eye Res. 10.1016/j.exer.2012.04.016 PMC3612883 22575566

[39] Clemons M, Goss P (2001) Estrogen and the risk of breast cancer. N Engl J Med 344: 276–285. 11172156 10.1056/NEJM200101253440407

[40] Ko Y, Abel J, Harth V, Brode P, Antony C, et al (2001) Association of CYP1B1 codon 432 mutant allele in head and neck squamous cell cancer is reflected by somatic mutations of p53 in tumor tissue. Cancer Res 61: 4398–4404. 11389067

[41] Hanna IH, Dawling S, Roodi N, Guengerich FP, Parl FF (2000) Cytochrome P450 1B1 (CYP1B1) pharmacogenetics: association of polymorphisms with functional differences in estrogen hydroxylation activity. Cancer Res 60: 3440–3444. 10910054

[42] Giulietti A, Overbergh L, Valckx D, Decallonne B, Bouillon R, et al (2001) An overview of real-time quantitative PCR: applications to quantify cytokine gene expression. Methods 25: 386–401. 11846608 10.1006/meth.2001.1261

