# Supplementary figures and images for: Molecular Basis for Involvement of CYP1B1 in MYOC Upregulation and Its Potential Implication in Glaucoma Pathogenesis
Source: PLoS One. 2012 Sep 21;7(9):e45077. doi: 10.1371/journal.pone.0045077 (PMC3448602; doi:10.1371/journal.pone.0045077)

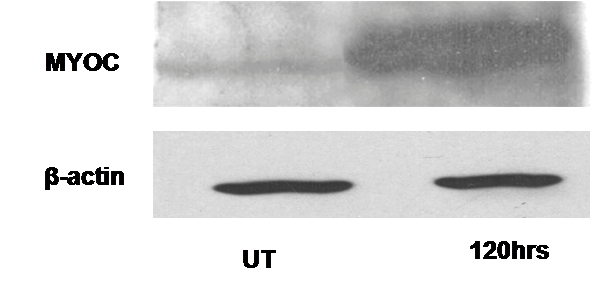


**Figure S1**

Supplement: Figure S1 — Upregulation of myocilin upon dexamethasone treatment. A considerable overexpression of myocilin in HTM cell line is observed upon treatment with 100 mM dexamethasone for 5 days (DOC) [file pone.0045077.s001.doc]
