# Supplementary material for: Molecular Basis for Involvement of CYP1B1 in MYOC Upregulation and Its Potential Implication in Glaucoma Pathogenesis
Source: PLoS One. 2012 Sep 21;7(9):e45077. doi: 10.1371/journal.pone.0045077 (PMC3448602; doi:10.1371/journal.pone.0045077)
Supplement: Table S1 — Primer sequences and PCR conditions for serial amplification of promoter regions in MYOC. (DOCX) [file pone.0045077.s002.docx]

**Table S1:**

**Primer sequences and PCR conditions for serial amplification of promoter regions in *MYOC***

| Primer Sequence | **cis-elements of interest present in the DNA fragment** | **Annealing temp. (^0^C)** | **Mg^2+^ conc. (mM)** | **Product size (bp)** |
| --- | --- | --- | --- | --- |
| *Forward primers (5’-3’)* |  |  |  |  |
| **TIGR45aF**-CCGCCTCGAGCCTACCTTCGTGGAGGTGAC | 2 AP1 & 2^1^/_2_ ERE site | 54 | 2.0 | 3194 |
|  |  |  |  |  |
| **TIGR45cF**-CCGCCTCGAGCAGCGATGTTTACTATCTG | 2 AP1 & ^1^/_2_ ERE site | 56 | 2.0 | 1449 |
|  |  |  |  |  |
| **TIGR45dF**-CCGCCTCGAGCAGTTGTTGCAGATACGTTGTAAG | 2 AP1 sites | 60 | 2.5 | 900 |
|  |  |  |  |  |
| **TIGR45eF**-CCGCCTCGAGGAAGAGTTCCCCAGATTTCACC | 1 AP1 site | 52 | 3.0 | 700 |
|  |  |  |  |  |
| *Common reverse primer for all 4 forward primers (5’-3’)* |  |  |  |  |
| TIGR45R-GCGCAAGCTTGCTGAGAGGTGCCTGGATGG |  |  |  |  |
