# Supplementary material for: Molecular Basis for Involvement of CYP1B1 in MYOC Upregulation and Its Potential Implication in Glaucoma Pathogenesis
Source: PLoS One. 2012 Sep 21;7(9):e45077. doi: 10.1371/journal.pone.0045077 (PMC3448602; doi:10.1371/journal.pone.0045077)
Supplement: Table S2 — Primers used for ChIP assay. (DOCX) [file pone.0045077.s003.docx]

**Table S2: Primers used for ChIP assay**

| **Primer name** | **Primer sequence (5’-3’)** | **Genomic region** | **Annealing temperature** | **Product size** |
| --- | --- | --- | --- | --- |
|  |  |  |  |  |
| TIGR19PF | GGTGAGCAAAGCAGTCACTG | Myocilin promoter | 58^o^C | 308bp |
| TIGR19PR | GGAGTCTCCAGCTCAGATGC |  |  |  |
|  |  |  |  |  |
| TYR-1PF | GCCATGAATTTCAGTTTCCCTA | Tyrosinase promoter | 58˚C | 539bp |
| TYR-1PR | CCTGATGCATTTTCTGACAACTCC |  |  |  |
|  |  |  |  |  |
