# Supplementary material for: Molecular Basis for Involvement of CYP1B1 in MYOC Upregulation and Its Potential Implication in Glaucoma Pathogenesis
Source: PLoS One. 2012 Sep 21;7(9):e45077. doi: 10.1371/journal.pone.0045077 (PMC3448602; doi:10.1371/journal.pone.0045077)
Supplement: Table S3 — Primers for site directed mutagenesis of CYP1B1 constructs. (DOCX) [file pone.0045077.s004.docx]

**Table S3**: **Primers for site directed mutagenesis of CYP1B1 constructs**

| **Primer Name** | **Primer Sequence (5’-3’)** |
| --- | --- |
| CYP-E229K-F | GCTGCTCAGCCACAACAAAGAGTTCGGGCGCAC |
| CYP-E229K-R | GTGCGCCCGAACTCTTTGTTGTGGCTGAGCAGC |
|  |  |
| CYP-R368H-F | GGTCGTGGGGAGGGACCATCTGCCTTGTATGGG |
| CYP-R368H-R | CCCATACAAGGCAGATGGTCCCTCCCCACGACC |
|  |  |
| CYP-R523T-F | GTCAATGTCACTCTCACAGAGTCCATGGAGCTC |
| CYP-R523T-R | GAGCTCCATGGACTCTGTGAGAGTGACATTGAC |
