# Supplementary material for: Molecular Basis for Involvement of CYP1B1 in MYOC Upregulation and Its Potential Implication in Glaucoma Pathogenesis
Source: PLoS One. 2012 Sep 21;7(9):e45077. doi: 10.1371/journal.pone.0045077 (PMC3448602; doi:10.1371/journal.pone.0045077)
Supplement: Table S4 — Primers used for RT-PCR to amplify genes in estrogen synthesis pathway. (DOCX) [file pone.0045077.s005.docx]

**Table S4: Primers used for RT-PCR to amplify genes in estrogen synthesis pathway**

| **Primer name** | **Primer sequence (5’-3’)** | **Gene** | **Product size (bp)** |
| --- | --- | --- | --- |
| SCC1F | TGGCTGAGCAAAGACAAGAA | P450SCC | 214 |
| SCC1R | AGGTGAAGGAGATGGGCTTT- |  |  |
|  |  |  |  |
| ARM1F | TGCAAAGCACCCTAATGTTG | Aromatase | 212 |
| ARM1R | TTGTCCCCTTTTTCACTGG |  |  |
|  |  |  |  |
| CYP17-F4 | ACCAGAATGTGGGTTTCAGC | CYP17A1 | 168 |
| CYP17-R4 | CCTTGTCCACAGCAAACTCA |  |  |
|  |  |  |  |
| HSD3B-F2 | ATCCACACCGCCTGTATCAT | HSD-3B | 208 |
| HSD3B-R2 | TTTCCAGAGGCTCTTCTTCG |  |  |
|  |  |  |  |
| HSD17B12-F | CCTGTCCCACTCTTGACCAT | HSD17B12 | 158 |
| HSD17B12-R | AAAGTTGGCTTCCGGATTTT |  |  |
|  |  |  |  |
